# Word sense disambiguation for event trigger word detection in biomedicine

**DOI:** 10.1186/1471-2105-12-S2-S4

**Published:** 2011-03-29

**Authors:** David Martinez, Timothy Baldwin

**Affiliations:** 1 NICTA VRL, The University of Melbourne, VIC 3010, Australia; 2 Department of Computer Science and Software Engineering, The University of Melbourne, VIC 3010, Australia

## Abstract

This paper describes a method for detecting event trigger words in biomedical text based on a word sense disambiguation (WSD) approach. We first investigate the applicability of existing WSD techniques to trigger word disambiguation in the BioNLP 2009 shared task data, and find that we are able to outperform a traditional CRF-based approach for certain word types. On the basis of this finding, we combine the WSD approach with the CRF, and obtain significant improvements over the standalone CRF, gaining particularly in recall.

## Introduction

In recent years, the biomedical text processing field has created many annotated resources to further the development of automatic text analysis methods through standardised evaluation. Following the example of TREC [1] and other efforts, the goal is to develop datasets over which different approaches can be tested and compared. There is considerable diversity in the types of resources that have been produced, from TREC-style document relevance scores to the semantic annotation of all terms in a set of documents (for entities and events of interest).

Different resources annotate data at varying levels of abstraction. In some cases, the target concepts are named entity mentions, such as names of proteins or medications, on which traditional named entity recognition (NER) techniques perform well [2]. In other cases, however, the annotation is over biomedical processes such as events, and the task has been found much harder to tackle [3]. The main problem is that a single class is used to annotate a wide range of linguistic realisations, and NER approaches that rely on recurrences of tokens with predictable labelling are often tripped up. For instance, consider the following examples from the BioNLP 2009 event extraction task [4], where the tags are shown as sub-indices to the open brackets of the target words:

(1) The [_TRANS_ changes] in the mRNA levels of these protooncogenes...

(2) The human platelet-activating factor receptor (PAFR) gene is [_TRANS_ transcribed] by...

We can see that two instances have been tagged with the event *TRANS* (short for TRANSCRIPTION), which refers to the process of creating an equivalent RNA copy of a sequence of DNA. This event is associated with different surface forms in the text: the noun *changes* in (1) and to the verb *transcribed* in (2). These types of wide-ranging lexical variations are common across all event types in the BioNLP task, and make both the annotation and the task challenging, with the best system in the shared task obtaining just above 50% F-score in the basic event structure recognition task (task 1: [4]).

When studying the BioNLP 2009 dataset, we observed that there is a set of high-frequency word types that tend to occur over many event types. This suggests the possibility of building separate models for each of these word types, in a fashion applied in word sense disambiguation (WSD). This technique has not been tested by previous trigger-word detection systems, where the approach is to build event-centred systems, or rely on hand-made dictionaries. The appeal of WSD is that it has been studied widely, and has been shown to perform well under certain conditions: shared tasks like SemEval [5] have illustrated that WSD systems can perform above 70% accuracy for fine-grained sense inventories [6]; and the performance over NLM-WSD, a collection tailored to the biomedical domain, is close to 90% accuracy [7]. Should we be able to replicate these levels of accuracy over the BioNLP 2009 data, it has the potential to boost overall trigger word detection performance.

Another motivation for combining biomedical datasets and WSD is that the WSD community is constantly looking for new semantically-annotated data to test the portability of their systems over. There are only a few examples of domain-specific corpora annotated with sense information, and they are costly to produce [8]. For instance the NLM-WSD collection [9] was constructed using 11 annotators, and has been used extensively for biomedical WSD experiments. An alternative could be to adapt the semantic annotation of BioNLP and other related biomedical datasets (e.g. BioCreative [10]) in order to generate more testbeds.

To summarise, our main hypothesis in this paper is that biomedical term-annotation tasks can benefit from WSD methods, which we will empirically test over the BioNLP event extraction dataset, where the correct identification of “trigger words” is a crucial component of the overall problem. We will adapt this dataset into a WSD-style collection, and evaluate the performance over a sample of word types that have particular properties, such as having enough training examples and a class distribution that is not overly skewed. We will analyse the raw performance scores to see if we can achieve similar performance to those achieved over other WSD problems, and we will also study the dataset itself, by measuring the strength of the “one sense per collocation” heuristic in relation to other WSD datasets.

The primary findings of this paper are: (a) WSD can indeed outperform sequential tagging techniques over high-frequency terms with relatively low skew; and (b) the overall performance of sequential tagging methods is boosted when we selectively include predictions from our WSD model.

This article is organised as follows. We describe the background of our research in Section . We then introduce our experimental setting in Section , and perform an analysis of feature types in Section . After this study, our main experimental results are presented in Section . We discuss further our experiments and analyse the errors in Section , and finally present our conclusions and future directions in Section .

## Background

In this paper, we focus exclusively on the BioNLP 2009 shared task dataset, where events are defined relative to trigger words of different types, and the goal is to both identify the trigger words and infer the role they play in a given event. There are three separate subtasks in this challenge, and in this paper we focus on Task 1: Core event extraction. Trigger words are linked to a total of 9 events relating to protein biology, which we list in Table 1. The annotation of protein occurrences in the text is given to participants in advance, and these are used as arguments for event triggers.

**Table 1:** List of target events for BioNLP 2009

Gene expression
Transcription
Protein catabolism
Localization
Binding
Phosphorylation
Regulation
Positive regulation
Negative regulation

In the original BioNLP 2009 shared task, most systems relied on at least two separate modules: trigger detection and event construction. Trigger detection involves the identification of event triggers and their type, while event construction associates event triggers with their arguments. For our experiments, we will focus on the trigger detection task, in order to simplify the analysis and comparison of different methods. The systems present in the BioNLP shared task addressed the trigger detection subtask by relying on hand-made dictionaries, sequential classifiers, or class-specific models; not word type models as we do in this paper. The top-ranked system in the 2009 BioNLP shared task was developed by a team from the University of Turku [11]. Their pipeline consists of three main steps: trigger detection, argument assignment, and semantic post-processing. For trigger detection they treat each token as a separate classification problem, and train SVMs for each event type. They rely on a rich set of features, including a dictionary built from the training data, and syntactic dependencies. Their overall task-1 system achieved an F-score of 52% (with 47% recall and 58% precision) by relying on separate SVM classifiers for trigger detection and argument assignment. However, the performance over the trigger detection step in isolation was not reported.

The second-ranked system for the task was built by a team from the University of Jena [12]. Their main architecture also had an independent module for trigger detection, which relied heavily on hand-curated dictionaries, built from the GENIA event corpus [3] and other sources. Their work required manual effort to pre-identify the predictive power of candidate trigger words for each event type, and they relied on this information to build dependency graphs that were refined in subsequent steps. The recall of their system was similar to the top ranked system, but the precision was much lower. Again, their performance for trigger word detection is not known.

We turn to the WSD literature to see if these techniques can contribute to the trigger detection task. There is a large literature on WSD; see [13] and [14] for recent overviews. The most successful approaches are supervised systems that build a separate model for each word type and POS, learning only from contexts that include it. The motivation behind this approach is the “one sense per collocation” heuristic [15], which observes that the meaning of a given word in a particular collocation tends to be invariant across all token occurrences.

One method that has been shown to perform consistently well over open-domain WSD and biomedical WSD is the Vector Space Model (VSM). It achieved high performance over the biomedical NLM-WSD collection (close to 90% accuracy) [7], and it has also been applied to the Senseval-3 English Lexical Sample dataset [16], where it ranked among the top systems with an accuracy of 72% [17]. This classifier can accommodate a wide range of features, from local dependencies to MeSH terms (cf. Section ).

## Experimental setting

We designed an experiment to integrate a WSD module into an event-extraction system based on the BioNLP 2009 shared task data. We first describe the dataset used in this experiment, then the different systems tested.

### Datasets

In order to build a WSD collection, we first identified the candidate target words in the BioNLP data that are most likely to benefit from WSD, in terms of both WSD having a good chance of performing well over them, and there being enough token instances that, when fed back into a larger system, the predictions can potentially have a significant impact. Each word occurrence in the data will have one of the 9 trigger-word event classes or the non-event class (a total of 10 classes). Candidate word types for WSD are those which have high frequency and occur with different event classes. We rely on the GENIA tagger [18] for tokenisation and POS-tagging, and we group word occurrences by lemma and POS.

For this experiment we chose the words in the BioNLP 2009 training data that had a token bias lower than 90% for the majority class (i.e. there are more than 10% of token instances which occur with a class other than the majority class), and at least 50 training instances. We separate the occurrences of words according to their POS, e.g. we would separate out the noun and verb instances of the word *change*, and build dedicated WSD classifiers for each. The 90% threshold filters out word types that have a strong prior for a single class, where there is little margin for improvement. Keeping these high-skew words would boost the performance, but we opted to remove them to focus on the higher-entropy, more interesting cases. The word selection process leaves us with 63 word types, which correspond to 14,903 train instances and 2,910 test instances. Note that we use the development data provided by the task organisers as the test set, since the final test data has not been released. Out of these test instances, 848 belong to annotated events, covering 65% of the 1,300 trigger event annotations in the test set. If we include the word types with high skew (i.e. 90% or more), we cover 990 trigger-annotated instances (76% of the test events), leaving only 24% of triggers to be identified with other techniques. The complete list of target words is given in Table 2. We also rely on existing WSD datasets in order to analyse if our new collection has significant differences regarding the class-entropy of the features. For this, we studied two WSD corpora where our WSD method has been shown to perform well: the biomedical NLM-WSD collection, and the open-domain Senseval-3 English Lexical Sample collection. The former consists of 50 word types with 100 annotated token instances each; the latter contains 57 word types, with an average of 132 annotated token instances each. Our own BioNLP dataset has an average of 282 token instances per word type.

**Table 2:** List of target words for our WSD experiment (N = noun, V = verb, J = adjective). We present the number of train and test instances, the number of classes, and the bias of the majority class.

Word	Train #	Test #	Classes	# Top class %
expression.N	1465	265	5	0.51
transcription.N	1214	210	2	0.83
activation.N	1177	272	3	0.89
promoter.N	770	141	3	0.76
binding.N	625	113	2	0.84
induce.V	565	128	3	0.65
activate.V	523	93	2	0.84
effect.N	416	79	4	0.83
inhibit.V	412	57	2	0.67
induction.N	405	116	4	0.57
bind.V	373	80	2	0.52
role.N	342	75	3	0.87
express.V	308	55	5	0.57
increase.V	289	59	2	0.57
stimulation.N	284	53	4	0.89
regulation.N	274	52	3	0.66
regulate.V	265	49	3	0.59
require.V	257	53	3	0.74
production.N	251	45	4	0.49
inhibition.N	219	38	2	0.79
mediate.V	218	54	3	0.74
stimulate.V	215	35	3	0.83
result.V	180	35	4	0.86
enhance.V	178	27	2	0.62
phosphorylation.N	170	52	3	0.64
increase.N	157	27	2	0.52
lead.V	146	25	2	0.83
interaction.N	144	41	2	0.71
associate.V	138	43	2	0.85
block.V	138	28	3	0.67
control.N	124	26	2	0.87
translocation.N	123	20	2	0.76
tyrosine.N	119	24	2	0.76
synthesis.N	119	9	3	0.75
detect.V	109	16	7	0.76
tNF.N	108	11	2	0.70
inducible.J	108	28	3	0.78
affect.V	107	14	4	0.62
transactivation.N	106	4	2	0.86
nucleus.N	104	12	2	0.85
decrease.V	98	12	2	0.54
reduce.V	90	26	2	0.52
control.V	89	17	2	0.65
suppress.V	89	20	2	0.65
degradation.N	88	15	2	0.72
produce.V	87	19	3	0.55
transcript.N	80	21	2	0.88
occur.V	80	24	2	0.89
target.N	75	16	3	0.87
dependent.J	73	16	3	0.71
cause.V	72	10	2	0.85
essential.J	70	9	2	0.84
interact.V	70	15	2	0.51
heterodimer.N	69	22	2	0.84
secretion.N	66	11	2	0.73
prevent.V	65	12	2	0.60
change.N	62	10	3	0.81
transfecte.V	61	25	3	0.85
absence.N	60	10	7	0.83
modulate.V	58	8	3	0.79
contribute.V	55	16	3	0.78
decrease.N	51	10	2	0.61
cross-linking.N	50	2	2	0.52

### Classifiers and features

Our main WSD classifier is based on the **Vector Space Model (VSM)**, in the form of a nearest-prototype classifier. Each occurrence of an ambiguous word is represented as a binary vector in which each position indicates the occurrence/absence of a feature, and a single centroid vector is generated for each sense of each word type during training. These centroids are compared with the vectors that represent new examples using the cosine similarity metric. The sense assigned to a given test instance is that of the closest centroid.

As a secondary WSD classifier we use a **Support Vector Machine (SVM-Weka)**, as implemented in the Weka toolkit [19]. SVMs map feature vectors into a high-dimensional space and construct a classifier by searching for the hyperplane in that space that gives the greatest separation between the classes. For our experiments we rely on a polynomial kernel, with the C parameter set to 1 (the default value in Weka). In both cases, we build a separate classifier for each word type.

Both WSD classifiers rely on an extensive set of features:

• *Local collocations* (*Local*): A set of features which describe the context of the ambiguous word token, in the form of: (1) bigrams and trigrams containing the ambiguous word constructed from lemmas, word forms, POS tags and PROTEIN tags provided in the BioNLP 2009 dataset; and (2) the lemma/word-form of preceding/following content words (adjectives, adverbs, nouns and verbs) occurring in the same sentence as the target word.

• *Syntactic dependencies*: We identify the syntactic dependencies between the target word and other words in the sentence. We define two types of features: (1) lexicalised dependencies (the dependency relation type + the related lexical item), and (2) unlexicalised dependencies (the dependency relation type only). We extract the dependencies from the four parsers provided by the BioNLP 2009 shared task organisers [20].

• *Bag-of-words* (*BOW*): Lemmas of all content words (nouns, verbs, adjectives, adverbs) in the same sentence as the target word, and, as a separate feature, lemmas of all content words within a ±4-word window around the target word.

• *Medical Subject Headings* (*MeSH*) [21]: the manually-assigned MeSH terms associated with the document that the sentence was taken from. MeSH is a controlled vocabulary for indexing biomedical and health-related information and documents, and all biomedical papers in MEDLINE are indexed with MeSH data.

We also implement a sequential tagger that does not follow the WSD approach of separate models for each word type. Specifically, we use the CRF++ toolkit [22], which has been shown to be highly successful over various chunking tasks. CRFs provide a discriminative framework for building structured models to segment and label sequence data [23]. CRFs have the well-known advantage that they both model sequential effects and support the use of large numbers of features.

For the CRF classifier we used a similar set of feature types to the WSD classifiers: word-forms, lemmas, POS, chunk tags, protein annotations, and grammatical dependencies. For dependency annotation, we used the Bikel parser and GDep as provided by the shared task organisers. This information was provided as a feature that expresses the grammatical function of the token. We applied a window size of 4 words in either direction from the target word.

Note that the CRF classifier is able to classify occurrences other than those for the target word types, but we evaluate only over the 63 target words for a fair comparison.

Finally, we also built an extended version of CRF (CRF-VSM) which uses the WSD predictions as an extra feature. In cases where the current token is none of the target words, the feature has the value NULL. For the training data, we obtain the predictions by using 3-fold cross-validation, and for the test data we rely on the full training set. This system allows us to combine the NER and WSD approaches to the problem. For evaluation we provide the precision, recall, and F-score for each class, in addition to reporting the micro-averaged results over the 9 trigger-word classes. We also show the average accuracy across all instances; this score is less relevant to the final goal of correctly identifying trigger words, because it is affected by the predominance of the NON-EVENT instances and ignores recall.

We use randomised estimation to calculate whether any performance differences between methods are statistically significant [24]. As a baseline we present the Majority Class (MC) classifier, which assigns the most frequent class seen in the training data to all the test instances.

## Feature analysis

Supervised WSD systems build upon the “one sense per collocation” heuristic, which shows that in fixed collocations, a given word will tend to occur with the same meaning. Yarowsky [15] defined “collocation” as the co-occurrence of words in a given relationship, for instance the words *no relevant* occurring immediately before the word *changes*. The features that we used satisfy this definition of “collocation”. We analyse the class entropy of the features in our collection and compare it with the WSD corpora introduced in Section . We rely on all features that occur at least twice, and we average the entropy values of all the features for each of the four basic feature types. We did not have access to all feature types for every corpus (e.g. MeSH is not found in Senseval), but the available ones can give us some insight into the differences across the corpora.

The average entropy per feature type and corpus are given in Table 3. Looking at the overall entropy for the BioNLP corpus, we can see that it falls between the low entropy of the domain-specific NLM and the higher entropy of Senseval. This is encouraging because our classifier has been able to obtain good performance over the Senseval dataset. The scores for the NLM collection show that this dataset is particularly well-suited to WSD, which has been illustrated by the high performance reported in the literature [7].

**Table 3:** Entropy for each feature type across the three WSD corpora

Feature type	BioNLP	NLM	Senseval
Local	0.301	0.176	0.380
Syntactic dep.	0.305	—	0.280
BOW	0.339	0.186	0.455
MeSH	0.360	0.183	—

Overall	0.323	0.180	0.435

With respect to the different feature types, there are not big differences in our collection, and as expected, local features and syntactic dependencies do best. More surprising are the results across Senseval data, with the high entropy of BOW and the low score of syntactic dependencies. NLM features have low entropy for all the different types.

We also analysed the entropy for the different target word types in the BioNLP dataset. We grouped the words into three sets based on their average feature entropy, as can be seen in Table 4, together with examples of word types. We expect that words with low entropy will achieve better performance, and we test this idea in Section.

**Table 4:** Number of word types and their average training frequency for different average entropy ranges. An example for each group is provided, together with its most frequent class.

Average Entropy	# Words	Avg. Freq.	Example	Major Class
*H* < .3	28	272.8	occur.V	NON-EVT
.3 ≤ *H* < .4	20	167.3	change.N	NON-EVT
*H* ≥ .4	15	264.8	express.V	GENE-EXP

## Results

We analyse the results of the different classifiers over the development portion of the BioNLP dataset. The scores of the four classifiers and the Majority Class (MC) baseline are given in Table 5. SVM-Weka performs poorly, just above the majority class baseline in terms of F-score. We can see that VSM suffers from low precision, and CRF has low recall, but both achieve an F-score quite a bit higher than SVM-Weka, despite SVMs having been found to be the strongest performer by the top two teams in the original shared task. The smaller set of instances used to build the models could be one of the reasons for the low performance of our SVM. By using only instances from the target word type, the training data for each class is reduced considerably, and the conceptually simpler VSM performs better in this setting. Another reason for the low performance of SVM could be the lack of tuning of the kernel and parameters. The best performer of all, however, in terms of both accuracy and F-score, is the combined CRF-VSM, where the output of VSM is incorporated as a feature into CRF. This approach significantly improves the recall and F-score of the base CRF (p-values < 0.01), and it is also significantly better in precision (p-value < 0.01) and F-score (p-value < 0.05) than the base VSM. That is, the combination of the two approaches performs better than each of the two methods in isolation. This result is highly significant in suggesting a new approach to the BioNLP 2009 task, or at the very least, a new source of features to combine with the existing task 1 systems.

**Table 5:** WSD performance of the different classifiers (the best results per column are given in bold)

System	Acc	Prec	Rec	F-score
MC	72.8	55.9	27.4	36.7
SVM-Weka	62.7	39.9	39.6	39.8
CRF	78.4	**72.4**	46.3	56.5
VSM	71.7	54.4	**62.5**	58.1
CRF-VSM	**78.9**	70.2	52.6	**60.1**

We also compared the performance of CRF and VSM according to the average entropy of the target words. The results are given in Table 6. We can see that VSM obtains the best performance for low-entropy words, and it is able to clearly improve over CRF for words with an average feature entropy below 0.40. The improvement in recall is statistically significant (*p* < 0.01), and the F-score improvement is statistically significant for words with entropy below 0.30 (*p* < 0.05). This demonstrates that we have the ability to pre-identify words that are most likely to benefit from WSD. Note also that, surprisingly, the best performance overall is obtained in the high-entropy range.

**Table 6:** WSD result for different entropy ranges (the best score for each evaluation metric and entropy range is shown in bold)

Average Entropy	CRF			VSM		
	Prec	Rec	F-sc.	Prec	Rec	F-sc.
*H *< .3	**71.6**	25.7	37.9	39.5	**58.3**	**47.1**
.3 ≤ *H *< .4	**76.5**	42.1	54.3	63.3	**56.1**	**59.5**
*H *≥ .4	**70.6**	60.9	**65.4**	59.9	**69.5**	64.3

We also report the results of CRF-VSM by event type in Table 7. We can see that different types exhibit very different performance, and the most difficult to predict are regulation events. This could happen because these three classes tend to occur in similar contexts, and are hard to differentiate. The best performance is achieved by the rare PROTEIN CATABOLISM event, with full precision and a high F-score of 91.7%; the high-frequency event GENE EXPRESSION ranks second with an F-score of 76.7%.

**Table 7:** Performance of CRF-VSM by event type, sorted by F-score (Freq. = Frequency of the event in test data).

Event	Freq.	Prec.	Rec.	F-score
PROTEIN CATABOLISM	13	100	84.6	91.7
GENE EXPRESSION	208	75.9	77.4	76.7
PHOSPHORYLATION	34	82.8	70.6	76.2
LOCALIZATION	13	72.7	61.5	66.7
BINDING	119	78.7	52.9	63.3
TRANSCRIPTION	52	64.0	61.5	62.7
POSITIVE REGULATION	263	64.9	42.2	51.2
REGULATION	86	51.2	25.6	34.1
NEGATIVE REGULATION	60	50.0	23.3	31.8

Finally, we present the overall results for each POS in Table 8. The main observation is that the F-score and recall are much higher for nouns than for verbs, but the precision and accuracy for the two are similar.

**Table 8:** WSD experiment performance of VSM by POS. The best result per column is given in bold.

POS	# Test	Acc	Prec	Rec	F-score
Noun	1802	**72.9**	**55.8**	**70.0**	**62.1**
Verb	1055	70.0	53.2	53.2	53.2
Adj.	53	64.2	9.1	10.0	9.5

The difficulty of disambiguating verb senses has been observed variously in the WSD literature, and these results seem to confirm that tendency. Regarding adjectives, the performance is actually below the majority class baseline, but there are only three word types and relatively few token instances for each, so the overall impact on results is negligible. The difficulty here appears to relate to the choice of which word to annotate as the trigger word, and a richer model of the interaction of the words related to the event would be required to improve the performance.

## Discussion

Our results over the BioNLP 2009 dataset show that the transformation of existing biomedical annotation into a WSD dataset can be challenging. One of the main differences over standard WSD is that the word-models tend to be biased towards the non-event class, and this can be problematic for the classifiers. For the noun *transcription*, e.g., 83% of the training instances are of type NON-EVENT. A possible solution could be to apply re-sampling techniques to build more balanced models [25].

Another issue is the difficulty of event annotation for humans, even when strategies to ensure quality are put in place. After a long process, the BioNLP event annotation enforced the following guidelines [3]: text-bound annotation (grounding all annotations to strings in text), single-facet annotation (keeping the viewpoint of annotation simple and very focused), and semantic typing (looking at types of entities for each event, and types of event for each entity to detect anomalies). The developers of the corpus explain that this process improved the inter-annotator agreement significantly, although they did not provide the numbers. They also describe how some high-frequency words can represent a wide variety of biomedical events depending on their related words in the context.

In our error analysis we found that meta-linguistic knowledge would be necessary to correctly identify some of the targeted events, and richer features than standard WSD sets would be required. For instance, let us consider the noun *transcription* and the event type TRANSCRIPTION, which refers to the process of creating an equivalent RNA copy of a sequence of DNA. The word occurs 201 times as the TRANSCRIPTION event, and 1,013 times as NON-EVENT in the training data. Looking at the features, they have an average entropy of 0.23, which puts this target word among the low-entropy words. However, for this word the F-score of VSM is slightly lower than CRF, and we can see that some of the NON-EVENT occurrences have similar features to the event occurrences. By analysing the examples, we found the following phenomena, which are not captured by our current model:

1. The event is described in the text as a process, and the annotators mark the word that culminates it, not the initiator. In this example, *lack* is annotated instead of *transcription*:

The *transcription* was initiated from one of three EBNA promoters, Qp: by contrast, both Cp and Wp were silent, thus resulting in the [_TRANS_*lack*] of EBNA2 mRNA.

2. The event is underspecified in the sentence, referring to an unspecified gene, and it is not considered relevant. In this example, *transcription* is not annotated:

OTF-1-enriched protein fractions did not affect DRA gene transcription although it functionally enhanced the *transcription* of another gene.

3. Multiple events occur simultaneously, and the annotation has to accommodate their relationship. In the following example the main event is a negative regulation event (NEG-REG) that affects a transcription event. The annotators seem to focus on the surface form of the NEG-REG event first (marking *destabilization*), and then annotate as TRANSCRIPTION the noun phrase that is directly related to it, choosing *preformed*, and ignoring the first mention of *transcription*:

Glucocorticoids are known to downregulate interleukin-1 beta production in monocytic cells by two different mechanims: direct inhibition of the gene *transcription* and [_NEG-REG_ destabilization] of the [_TRANS_*preformed*] interleukin-1 beta mRNA.

4. The noun *transcription* occurs multiple times referring to the same event, but only the first occurrence is tagged:

A 2-4-fold increase in IFN-beta promoter [_TRANS_*transcription*] was observed in Sendai virus induced extracts, and deletion of PRDI and PRDII elements decreased this induced level of *transcription*.

These examples illustrate that for the BioNLP 2009 dataset, there are meta-linguistic aspects of the annotation that have to be taken into account, and more consistency is required to close the gap between textual representations and the ultimate goal of biomedical pathways. Significant effort has been done in the annotation of the BioNLP dataset, but we believe that word-by-word analysis can provide better means to improve and extend this kind of tagging. It has been shown in the WSD evaluation tracks that the annotation of lexical-sample datasets is easier and produces better quality data than all-words datasets, and this could be translated to the annotation of biomedical events. We have seen in this corpus that 63 ambiguous word types cover 62% of the event annotations, and focusing on the instances of these words separately could be a better way to produce consistent annotation.

## Conclusions

We described a WSD-based method for detecting event trigger words in the BioNLP 2009 shared task data, and demonstrated that it attains superior performance than a traditional sequential tagging approach. The highest score is achieved when using the WSD predictions as features for a sequential tagger, which significantly improves the recall and F-score of the latter. We also observed that measuring the training class-entropy of features seems to be a good indicator of the kind of target word types that can improve over a sequential tagger.

Another result of this work is the identification of consistency issues in biomedical annotation, even when clear guidelines are provided. We found that a word-centered approach may help to find inconsistencies, specially given that a few target words seem to have high coverage of the trigger annotations. For future work we are planning to explore other challenges, such as BioCreative, and also to deploy full systems for the BioNLP shared task challenge, in order to directly compare against other systems.

## Competing interests

The authors declare that they have no competing interests.
